# Dental Tissue and Stem Cells Revisited: New Insights From the Expression of Fibroblast Activation Protein-Alpha

**DOI:** 10.3389/fcell.2019.00389

**Published:** 2020-01-21

**Authors:** Ronald B. Driesen, Petra Hilkens, Nick Smisdom, Tim Vangansewinkel, Yörg Dillen, Jessica Ratajczak, Esther Wolfs, Pascal Gervois, Marcel Ameloot, Annelies Bronckaers, Ivo Lambrichts

**Affiliations:** ^1^Faculty of Medicine, Hasselt University, Diepenbeek, Belgium; ^2^Laboratory of Morphology, Biomedical Research Institute, Hasselt University, Diepenbeek, Belgium; ^3^Department of Biophysics, Biomedical Research Institute, Hasselt University, Diepenbeek, Belgium

**Keywords:** stem cell, molar, tooth, apical papilla, collagen, vimentin, odontoblast

## Abstract

Fibroblast activation protein-α (FAPα) is a membrane protein with dipeptidyl-peptidase and type I collagenase activity and is expressed during fetal growth. At the age of adolescence, FAPα expression is greatly reduced, only emerging in pathologies associated with extracellular matrix remodeling. We determined whether FAPα is expressed in human dental tissue involved in root maturation i.e., dental follicle and apical papilla and in dental pulp tissue. The dental follicle revealed a high concentration of FAPα and vimentin-positive cells within the stromal tissue. A similar observation was made in cell culture and FACS analysis confirmed these as dental follicle stem cells. Within the remnants of the Hertwigs’ epithelial root sheath, we observed FAPα staining in the E-cadherin positive and vimentin-negative epithelial islands. FAPα- and vimentin-positive cells were encountered at the periphery of the islands suggesting an epithelial mesenchymal transition process. Analysis of the apical papilla revealed two novel histological regions; the periphery with dense and parallel aligned collagen type I defined as cortex fibrosa and the inner stromal tissue composed of less compacted collagen defined as medulla. FAPα expression was highly present within the medulla suggesting a role in extracellular matrix remodeling. Dental pulp tissue uncovered a heterogeneous FAPα staining but strong staining was noted within odontoblasts. *In vitro* studies confirmed the presence of FAPα expression in stem cells of the apical papilla and dental pulp. This study identified the expression of FAPα expression in dental stem cells which could open new perspectives in understanding dental root maturation and odontoblast function.

## Introduction

Fibroblast activation protein alpha (FAPα), also known as seprase, is a type II membrane serine protease which displays functional similarities with prolyl-cleaving peptidases (Chung et al., 2014). The human FAPα gene is located on 2q24.2 and shares 50% homology in amino acids with the dipeptidyl peptidase IV (DPP-IV) enzyme. FAPα promotes type I collagen degradation through its collagenase activity (Chung et al., 2014). During embryogenesis, FAPα expression is highly upregulated in mesenchymal tissues where it contributes to extracellular matrix remodeling (Niedermeyer et al., 2001). When reaching adolescence, FAPα expression is almost completely lost but it re-emerges in pathologies associated with active tissue remodeling such as myocardial infarction (Tillmanns et al., 2015), pulmonary fibrosis (Fan et al., 2016), and cancer (Wen et al., 2016). At the cellular level, re-expression is found during fibroblast differentiation (Tillmanns et al., 2015) and is linked to circulating levels of TGF-β1 and IL-1β leading to activation of the SMAD pathway (Tillmanns et al., 2015). Loss of FAPα activity impacts the migratory capacity of myofibroblasts but does not affect their proliferation rate (Teichgräber et al., 2015). Recently it has been shown that FAPα is highly expressed in human bone marrow mesenchymal stem cells (BMSCs) (Chung et al., 2014) where it stimulates cell migration via RhoA GTPase activity, independent of its peptidase function.

The discovery of another source of MSCs in dental pulp tissue of third molars (Gronthos et al., 2000) i.e., dental pulp stem cells (DPSCs), led to the identification of a variety of MSCs defined by specific locations in the developing tooth, and the surrounding dental tissue. These include stem cells from the apical papilla (SCAPs), periodontal ligament stem cells (PDLSCs), and dental follicle stem cells (DFSCs). All are classified as MSCs according to the criteria proposed by the International Society for Cellular Therapy (Dominici et al., 2006; Huang et al., 2009). Whether FAPα is expressed in dental stem cells and if it contributes to dental tooth development is currently not known.

In this study, we investigated FAPα expression in the dental follicle and apical papilla, tissues both involved in root formation, and within the dental pulp of third molars of young adolescents. Visualization of FAPα expressing stem cells provided new evidence for the epithelial mesenchymal transition (EMT) within remnants of the Hertwig’s epithelial root sheath (HERS) and shed new light on the histological organization of the apical papilla. In addition, FAPα expression was highly pronounced in odontoblasts of the dental pulp suggesting a role in dentinogenesis.

## Materials and Methods

### Isolation and Cell Culture of Stem Cells From Dental Pulp and Apical Papilla

Dental follicles (*n* = 4) and healthy normal human third molars were collected from patients (14–21 years old) at the Ziekenhuis Maas and Kempen, Bree and ZOL Genk with written informed consent and approved by the medical ethical committee of Hasselt University (protocol 13/0104U). The apical papillae (*n* = 5) and dental pulp (*n* = 6) were separated from the teeth and all tissues were collected in α-Minimal Essential Medium (Sigma-Aldrich, Overijse, Belgium) supplemented with 10% heat inactivated fetal calf serum (FCS) (Biochrom AG, Berlin, Germany), 2 mM L-Glutamine, 100 U/ml Penicillin and 100 μg/ml Streptomycin (Sigma-Aldrich). Stem cells were isolated via the explant method as described previously (Hilkens et al., 2013). Briefly, pieces of 1 mm^3^ were placed into a 6-well plate containing culture medium. Explants were cultured for 14 days allowing stem cells to grow out of the tissue at 37°C in a humidified atmosphere containing 5% CO_2_. Medium was changed twice a week. After 10 to 14 days, 80% to 90% confluency was reached and cells were sub-cultured. For all experiments, cells of passage one to three were used.

### Histological Analysis

Tissue samples were fixed in 4% paraformaldehyde overnight and routinely embedded in paraffin. After deparaffinization and rehydration, 7 μm sections were stained for collagen using either Masson’s Trichrome or Sirius Red staining. Toluidine blue staining was performed on semi-thin sections of araldite embedded tissue. Prior to staining, samples were fixed with 2% glutaraldehyde in 0.05 M cacodylate buffer, post-fixed in 2% osmium tetroxide and stained with 2% uranyl acetate in 10% acetone. Samples were dehydrated in series of graded acetone concentrations and embedded in araldite according to the pop-off method.

### Immunohistochemistry and Immunocytochemistry

Antigen retrieval was performed in deparaffinized tissue sections using citrate buffer (Dako, Glostrup, Denmark) heated in the microwave oven (3 × 5′ cycli). After cooling down for 20′, sections were washed in phosphate buffered saline (PBS) and used for either diaminobenzidine (DAB) or fluorescent immunostaining. For DAB immunostaining, sections were treated with peroxidase block (Dako) for 20′. Afterward sections were washed with PBS and incubated with protein block (Dako) to limit background staining. Consequently, sections were incubated with a primary antibody against FAPα (1:200, Abcam, Ab2844), vimentin (1:100, Abcam, Ab8069), dsPP (1:200, Abcam, Ab216892) diluted in PBS for 1 h at room temperature followed by 3 washes with PBS. As negative control, the primary antibody was omitted from a section. Peroxidase-conjugated secondary antibodies diluted in PBS were applied for 45′ at room temperature followed by 3 washes in PBS. The chromogenic substrate DAB was used to visualize the protein of interest (DAB kit, Dako). Cells were counterstained with Mayer’s hematoxylin and mounted using DPX (Dibutylphthalate Polystyrene Xylene) mounting medium. The immune-reactivity was determined using a photomicroscope equipped with an automated camera (Nikon Eclipse 80i, Nikon Co., Japan).

For immunofluorescent staining, sections were treated with protein block, followed by a wash in PBS and incubation with primary antibodies against FAPα (1:200, Abcam, Ab2844), vimentin (1:100, Abcam, Ab8069), E-cadherin (1:200, Abcam, Ab231303), CXCR4 (1:50, Abcam, Ab124824), α-SMA (1:250, Thermo Fisher Scientific, asm-1), and CD44 (1:100, Abcam, Ab194987) overnight in a humidified atmosphere. As negative control, the primary antibody was omitted from a section. The next day, sections were washed with PBS and incubated with fluorochrome conjugated secondary antibodies for 1 h. After 3 washes in PBS, nuclei were counterstained with DAPI for 30 min and sections were mounted in fluorescent embedding medium (Dako). Fluorescent signal was imaged using a Leica fluorescence microscope (DM 4000 B LED) with the Leica Application Suite X software.

### Fluorescent Activated Cell Sorting Analysis

Stem cells were seeded in 25 cm^2^ culture flasks and were harvested by trypsinization after 7 days. Cells were incubated for at least 1 h at room temperature in PBS with 2% FCS to allow re-expression of receptor proteins at the cell surface. For intracellular staining of vimentin (1:100, Millipore) and nestin (1:100, Dako), cells were first fixed and then permeabilized with the cytofix/cytoperm kit (Becton–Dickinson, San Jose, CA, United States) according to manufacturer’s protocol. 0.5 × 10^5^ cells were washed with PBS containing 2% FCS and were incubated for 30 min at room temperature with primary antibodies against either CD24-PE (1:20, eBioscience, San Diego, CA, United States), CD31-PE (1:100, ImmunoTools), CD34-PE (1:100, ImmunoTools), CD44-PE (1:100, Abcam), CD45-PE (1:100, eBioscience), CD90-PE (1:100, eBioscience), CD105-PE (1:100, Abcam), and p75-PE (1:100, Dako). As a negative control for non-specific background staining, appropriate isotype controls were included. Thereafter, cells were washed three times with PBS and if necessary, incubated with secondary antibodies including FITC-labeled goat anti-rabbit (eBioscience) or PE-labeled anti-mouse IgG (Invitrogen) for 45 min at room temperature. Samples were analyzed on a FACScalibur^TM^ flow cytometer equipped with CellQuest software (BD Biosciences, Erembodegem, Belgium).

### Second Harmonic Generation and Confocal Microscopy

Second harmonic generation imaging was performed using a Zeiss LSM 880 (Carl Zeiss, Jena, Germany) mounted on an Axio Observer and equipped with a 20× objective (Plan-Apochromat 20×/0.8, Carl Zeiss) (Sanen et al., 2016). A pixel size of 0.69 μm was used with an image resolution of 1024 by 1024 and a pixel dwell time of 8.19 μs. Full tissue sections were imaged by means of tile scans with 10% overlap to enable the stitching of the recorded tiles. Excitation was provided by a femtosecond pulsed laser (MaiTai DeepSee, Spectra-Physics, CA, United States) tuned to a central wavelength of 810 nm. This laser was directed to the sample using a 760 nm shortpass dichroic mirror. The SHG signal and autofluorescence were collected in backward non-descanned mode using a 760 nm short pass dichroic mirror. A BP 350–690 bandpass filter was used to block any scattering infrared light. Finally, a 425 nm dichroic mirror separated SHG from autofluorescence which were subsequently simultanously recorded via, respectively, a 400–410 nm bandpass filter and a 450–650 nm band pass filter, by means of GaAsP detectors (BIG2, Carl Zeiss).

Confocal microscopy was performed using the same system as used for SHG imaging. Fluorescence was collected using a 63× (Plan-Apochromat 63×/1.4 Oil, Carl Zeiss). The resulting pixel size was 0.09 μm with a pixel dwell time of 2.65 μs. 3 laser lines were used for excitation: 488 nm (Ar-ion), 543 nm (HeNe) and 740 nm (MaiTai DeepSee, Spectra-Physics, CA, United States). A 488/543 nm dichroic and a 690 nm shortpass dichroic were used as main beam splitters. The resulting fluorescence was collected using a spectral GaAsP detector selecting 3 different spectral bands: 472–543 nm, 552–695 nm, and 414–472 nm.

### Statistical Analysis

Statistical analysis was performed using a Kruskal Wallis one-way ANOVA (non-parametric) on *n* = 4 independent cell cultures from 4 different donors. *p* < 0.05 is considered as statistically significant.

## Results

### Identification of Fibroblast Activation Protein-α Expressing Stem Cells in the Dental Follicle

**FIGURE 1:**
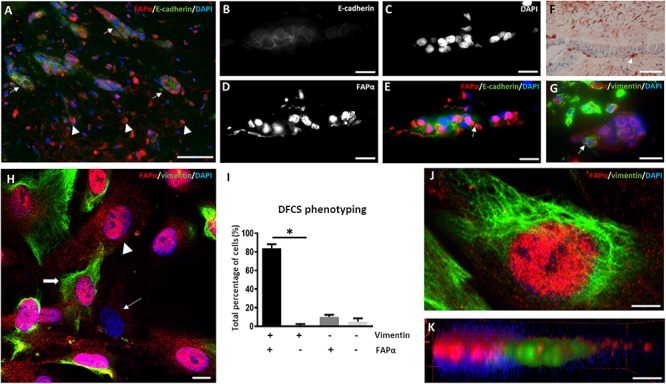
FAPα is expressed in stem cells of the dental follicle. **(A)** Double immunofluorescent staining in dental follicle tissue of FAPα (red), E-cadherin (green), and the nucleus (DAPI, blue). Arrows indicate epithelial rests and arrowheads point to FAPα^+^/E-cadherin^–^ cells. **(B–E)** Higher magnification of an epithelial rest displaying E-cadherin **(B)**, nuclei **(C)**, FAPα **(D)**, and the corresponding overlay **(E)**. **(F)** Visualization of mesenchymal cells in the dental follicle using vimentin staining. Note the presence of a vimentin positive cell at the periphery of the epithelial rest (white arrow). **(G)** Double immunofluorescent staining of FAPα and vimentin showing FAPα^+^/vimentin^–^ cells in the epithelial rest. A FAPα^+^/vimentin^+^ cell is located at the periphery of the epithelial rest. **(H)** Triple immunofluorescent staining of vimentin (green), FAPα (red), and nuclei (blue) in cultured dental follicle stem cells. Indicated cells are FAPα^+^/vimentin^+^ (thick arrow), FAPα^+^/vimentin^–^ (arrowhead), and FAPα^–^/vimentin^–^ (thin arrow). **(I)** Quantification of the different phenotypes based on vimentin and FAPα expression (*n* = 4; ^∗^*p* < 0.05). **(J)** Confocal image of triple immunofluorescent staining of vimentin (green), FAPα (red), and nuclei (DAPI). **(K)** 3-D volume rendering of **(J)** showing localization of FAPα on the plasma membrane. Scale bars represent 4 **(J,K)**, 10 **(B–E,G,H)**, and 50 **(A,F)** μm.

The dental follicle determines the growth of the periodontal ligament and the cementum and contains HERS remnants which promote dental root formation (Itaya et al., 2017). Immunofluorescent staining of the dental follicle revealed a high number of stromal cells (Figure 1A) which were positive for FAPα but negative for E-cadherin, an epithelial cell adhesion marker. This was contrary to the epithelial rests originating from HERS showing E-cadherin staining between intercellular connections (Figures 1B–E). FAPα staining was markedly present in a fraction of the epithelial cells and often co-localized with the nucleus (Figures 1C–E). The majority of epithelial cells stained negative for vimentin as demonstrated by immunohistochemistry (Figure 1F) and immunofluorescent (Figure 1G) staining. However, FAPα positive and vimentin positive cells were encountered at the periphery of the epithelial islands (Figures 1F,G). Next, we investigated FAPα and vimentin expression in cell cultures of DFSCs (Figures 1H,I). 84 ± 4% of the cells stained positive for FAPα and vimentin (Figure 1H, thick arrow) which was significantly higher than the vimentin positive but FAPα negative cells (*p* < 0.05). A total of 15% of the cells were negative for vimentin from which 10 ± 2% stained positive for FAPα (Figure 1H; arrowhead) and 5 ± 3.5% were FAPα negative (Figure 1H; thin arrow). The cellular localization of FAPα was determined by 3-D volume rendering of confocal z-stack images (Figure 1J) showing a diffuse distribution on the plasma membrane and/or a high concentration above the nucleus (Figure 1K). Flow cytometry of DFSCs revealed positive expression of the stem cell markers CD44, CD90, and CD105 in almost all cells (Supplementary Figure 1) but a lack of expression of CD31, CD34, and CD45. Based on these criteria, the majority of FAPα positive/vimentin positive cells are considered DFSCs.

### Fibroblast Activation Protein-α Expression in the Apical Papilla

**FIGURE 2:**
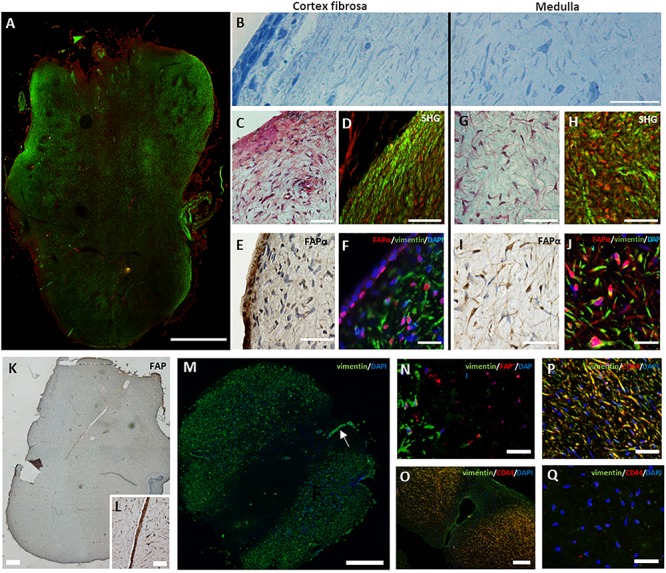
Heterogeneous expression of FAPα in the apical papilla. **(A)** Tile scan of a second harmonic generated overview of a complete apical papilla (green, collagen type I; red, autofluorescence). **(B)** Semi-thin section of an apical papilla stained with toluidine blue showing a parallel orientation of cells at the cortex fibrosa but random orientation inside the medulla. **(C)** Masson’s Trichrome staining of the cortex fibrosa showing a high collagen concentration. **(D)** Second harmonic generation imaging of the periphery of the apical papilla (green, collagen type I; red, autofluorescence). **(E)** DAB staining for FAPα reveals a heterogeneous expression pattern at the periphery of the tissue. Note the marked staining of FAPα in the epithelial region. **(F)** Double immunofluorescent staining showing predominantly vimentin positive cells (green) and a small population of FAPα^+^ (red)/vimentin^+^ cells. The epithelial region consists of FAPα^+^ but vimentin^–^ cells. **(G)** Masson’s Trichrome staining of the medulla reveals a loose extracellular matrix with spindle-shaped cells. **(H)** Second harmonic imaging confirms the loose collagen distribution (green). **(I)** DAB staining indicates high expression levels of FAPα in the medulla. **(J)** Immunofluorescent staining reveals a predominant population of FAPα^+^ (red)/vimentin^+^ (green) cells. **(K)** FAPα expression in the dental nerve (**K**, magnification in **L**). **(M)** Immunofluorescent staining showing absence of vimentin staining in the inner medulla. Note the presence of the vimentin positive nerve (**M**, arrow). **(N)** Double immunofluorescent staining showing vimentin negative but FAPα positive cells (red) in the inner medulla. **(O–Q)** Double immunofluorescent staining of vimentin (green) and CD44 (red) reveals double positive cells in the outer medulla (yellow) but complete absence of staining in the inner medulla. Scale bars represent 25 **(F,J,O,P,Q,N)**, 50 **(B,C,D,E,G,H,I,L)**, 100 **(J)**, 250 **(K,M)**, and 1000 **(A)** μm.

Third molars of young adolescents have a well-developed apical papilla at the base of the developing root (Sonoyama et al., 2008). Visualization of collagen type I using SHG microscopy identified two different morphological regions in the apical papilla (Figure 2A); the periphery with dense collagen type I and the inner tissue composed of less compacted collagen type I. Semi-thin sections indicated a different cellular orientation within these two regions (Figure 2B); (1) the periphery is composed of a single layer of cube-shaped epithelial cells and a sub-epithelial region containing cells with a parallel orientation and (2) the inner region of the apical papilla with randomly oriented cells. Masson’s Trichrome staining (Figure 2C), Sirius red staining (Supplementary Figure 2A) and SHG imaging (Figure 2D) confirmed that cells in the periphery are embedded in a tightly organized extracellular matrix displaying a parallel orientation. We defined this region as the cortex fibrosa. Immunohistochemistry showed the presence of FAPα positive cells within the epithelial layer and a lower density of FAPα positive cells in the sub-epithelial region (Figure 2E). Double immunofluorescent staining for FAPα and vimentin (Figure 2F) confirmed the DAB staining pattern of FAPα and demonstrated absence of vimentin staining in the epithelial layer. The majority of sub-epithelial cells in the cortex fibrosa are vimentin positive with a minority of cells expressing FAPα. The cortex fibrosa encapsulates the inner center which is composed of loose connective tissue with randomly distributed cells as shown by Masson’s Trichrome (Figure 2G) and Sirius Red (Supplementary Figure 2B) staining. SHG imaging confirmed the loose organization of collagen fibers (Figure 2H). This inner region which we defined as medulla contained a high concentration of FAPα and vimentin positive cells as shown by immunohistochemistry (Figure 2I) and double immunofluorescent staining (Figure 2J). Alpha-smooth muscle actin (α-SMA) staining was only restricted to the vasculature (data not shown). Serial sections of the medulla revealed FAPα positive staining in the central peripheral nerve which extends from the dental pulp through the apical papilla (Figures 2K,L). Surprisingly, immunofluorescent staining identified round-shaped cells in the inner part of the medulla and in the vicinity of the central nerve which were vimentin negative (Figures 2M,N) but still FAPα positive. Additional phenotypical characterization demonstrated that the vimentin expressing cells in the surrounding outer medulla were positive for CD44, a mesenchymal stem cell marker (Figures 2O–Q) whereas the vimentin negative fraction inside in the inner medulla stained negative for CD44 (Figures 2O,Q). Staining for cytokeratin 19 (data not shown), an epithelial cell marker, revealed no positivity within the vimentin negative cell population. Lastly, we observed FAPα staining within a palisade of columnar shaped cells bordering a small deposit of dentin indicating the presence of odontoblasts (Supplementary Figures 3A,B). Within the newly formed dentin, FAPα staining is visualized in small foci suggesting cytoplasmic extensions of odontoblasts in dentinal tubules (Supplementary Figure 3B, arrows).

### Molecular Characterization of Stem Cells From the Apical Papillas in Culture

**FIGURE 3:**
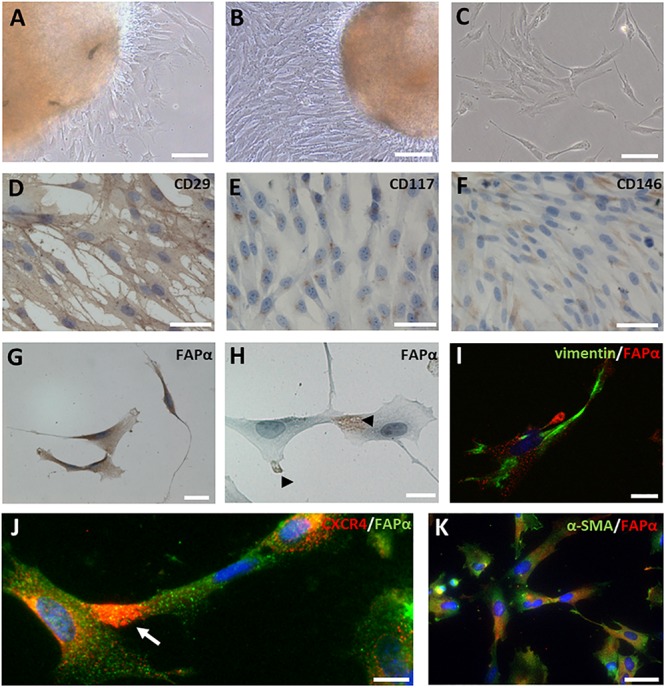
FAPα expression in SCAPs in culture. **(A–C)** SCAPs, isolated via the explant method, migrated out of the tissue explants, and displayed a spindle-shaped morphology. Cells were able to form colonies *in vitro* **(C)**. **(D–H)** Immunocytochemical staining of SCAPs for mesenchymal markers CD29, CD117, CD146, and FAPα. All cells expressed CD29 **(D)** and CD117 **(E)**. A subpopulation of the cells stained positive for CD146 **(F)**. FAPα staining was prominent in all cells and was visible on either the plasma membrane **(G)** or on pseudopodia **(H)**. **(I)** Double immunofluorescent staining **(I–K)** shows that FAPα positive SCAPs (red) co-express vimentin (**I**, green). **(J)** CXCR4 staining (red) was observed in pseudopodia (arrow) of FAPα expressing cells (green). **(K)** Diffuse α-SMA was observed in FAPα^+^ SCAPs (green). Nuclei are counterstained with DAPI (blue). Scale bars represent 20 **(H,I,K)**, 40 **(G)**, 50 **(D–F,J)**, 100 **(A–C)** μm.

After tissue isolation, SCAPs migrated out of tissue explants after 24 to 48 h in culture (Figure 3A) and reached confluency after 10 to 14 days (Figure 3B). The majority of SCAPs displayed a spindle-shaped morphology (Figure 3C). To evaluate mesenchymal marker expression, immunocytochemistry and FACS analysis were performed. SCAPs stained uniformly for the surface marker CD29 (Figure 3D) and a perinuclear expression pattern of CD117 (c-kit) was observed in all SCAPs (Figure 3E). A subpopulation of cells showed positive immune-reactivity for CD146 (Figure 3F). As shown in the Supplementary Figure 4, FACS revealed >95% expression of the mesenchymal stem cell markers CD44, CD90 and CD105, while the expression of hematopoietic stem cell markers CD34 and CD45 were absent (Supplementary Figure 4). A SCAPs subpopulation showed immune-positivity for p75 (10.3 ± 3.9%) but no expression of CD24. The majority of cells were positive for vimentin and nestin but SCAPs did not stain for CD31, an endothelial cell marker. Immunocytochemistry showed the presence of FAPα staining mainly on the plasma membrane (Figure 3G) but also in the nuclear region and in pseudopodia (Figure 3H). Double immunofluorescent staining indicated that the majority (70 ± 3%, *n* = 5 different donors) of vimentin positive SCAPs were FAPα positive (Figure 3I). CXCR4 which is involved in cell migration often co-localized with FAPα in the pseudopodia (Figure 3J). The majority of the SCAPs revealed a diffuse α-SMA staining which was absent in tissue sections (Figure 3K).

### Fibroblast Activation Protein-α Expression in Dental Pulp Tissue and Cultured Stem Cells

**FIGURE 4:**
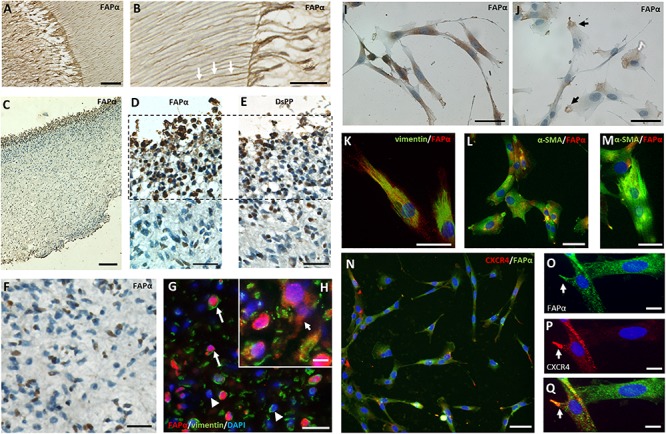
FAPα expression in dental pulp tissue and stem cells. **(A)** Immunohistochemistry staining of FAPα in a decalcified intact molar showing the dentin-pulp region. **(B)** Higher magnification displays FAPα^+^ odontoblasts with their corresponding protrusions entering the dental tubules. **(C)** FAPα staining of extracted pulp tissue. **(D)** Serial sections demarking the presence of FAPα **(D)** and DSPP **(E)** positive odontoblasts at the periphery of the extracted pulp tissue. **(F)** Immunohistochemistry staining of FAPα in the coronal center of extracted pulp tissue. A heterogeneous population of FAPα expressing cells is visible. **(G,H)** Double immunofluorescent staining of FAPα (red) and vimentin (green) with nuclear counterstaining (DAPI, blue) in matched dental pulp tissue. Indicated cells are FAPα^+^/vimentin^+^ (arrow) and FAPα^–^/vimentin^+^ (arrowhead). Insert **(H)** showing FAPα + staining in the plasma membrane (arrow). **(I)** Immunocytochemistry staining of FAPα in cultured DPSC’s showing localization in the plasma membrane (I) or limited to the pseudopodia **(J)**. Double immunofluorescent staining of cultured DPSCs indicates co-expression of FAPα with either vimentin (**K**, green) or diffuse α-SMA (**L**, green). Only a limited number of cells have organized α-SMA stress fibers **(M)**. **(N–Q)** Double immunofluorescent staining of FAPα (green) and CXCR4 (red) with nuclear counterstaining (DAPI, blue). Co-localization of FAPα **(O)** and CXCR4 **(P)** with the matching overlay image **(Q)**. Scale bars represent 25 **(B,D,E,F,H,K,M,O–Q)**, 50 **(A,I,J,L,N,G)**, and 100 **(C)** μm.

Immunohistochemistry of a third molar with intact pulp demonstrated FAPα staining in odontoblasts at the dentin-pulp complex (Figure 4A). Higher magnification showed FAPα positive protrusions of odontoblasts extending into dental tubules (Figure 4B; white arrows). In extracted dental pulp, odontoblasts were located at the periphery of the tissue and stained positive for FAPα (Figures 4C,D) and the specific odontoblast marker dentin sialophosphoprotein (DSPP) (Figure 4E). Immunohistochemistry revealed heterogeneous staining of FAPα within the center of the dental pulp (Figure 4F). We identified a cell population with FAPα- and vimentin-positive staining (Figure 4G, white arrow) pointing to a possible resident stem cell population and a second FAPα negative/vimentin positive cell population (Figure 4G, white arrowhead). FAPα staining was not only noted in the nuclei but also in the plasma membrane (Figure 4H, white arrow). α-SMA staining (images not shown) was restricted to blood vessels disproving the presence of FAPα and α-SMA positive myofibroblasts within the dental pulp. Immunocytochemistry of cultured DPSCs revealed FAPα staining on the plasma membrane (Figure 4I) or in the pseudopodia (Figure 4J). FAPα expressing DPSCs had vimentin positive intermediate filaments (Figure 4K) but diffuse α-SMA staining (Figure 4L). Only few cells displayed α-SMA positive stress fibers (Figure 4M) indicating a limited amount of myofibroblasts. The majority of FAPα positive DPSCs showed CXCR4 staining in their cytoplasmic extensions (Figure 4N) which frequently co-localized with FAPα (Figures 4O–Q).

## Discussion

In this study, we investigated the location and role of FAPα expression in dental tissues i.e., dental follicle, apical papilla and dental pulp and in their corresponding stem cells. First, we revealed that the dental follicle contains a rich source of FAPα positive cells which we identified as DFSCs. Within the HERS fragments, epithelial cells were observed showing FAPα expression. HERS cells undergo EMT in the presence of TGF-β1 (Akimoto et al., 2011) which is associated with HERS fragmentation and reduction in E-cadherin expression. Since FAPα expression is regulated by TGF-β1 (Tulley and Chen, 2014), we suggest that FAPα positive epithelial cells in HERS remnants could be part of a TGF-β1 driven EMT process. Collagenase type I activity exerted by FAPα could promote disintegration of the epithelial sheath enabling cell migration. At the periphery of HERS remnants, we observed FAPα and vimentin positive cells which suggests migration of newly differentiated mesenchymal cells into the extracellular matrix. However, we should not exclude the possibility that migration of FAPα positive/vimentin positive DFSCs into the epithelial sheath could cause epithelial degradation (Hirata and Nakamura, 2006).

Numerous studies have focused on the multi-lineage differentiation capacity of SCAPs but detailed knowledge on the histological organization of the apical papilla is lacking. We identified two novel histological regions with different features of FAPα expression. First, the medulla is the largest area of the apical papilla and contains a high concentration of FAPα, vimentin and CD44 positive cells corresponding to SCAPs in culture. Since FAPα exerts a collagenase type I activity, it can be hypothesized that the presence of FAPα in the medulla is associated with a continuous remodeling of the apical papilla during root formation. Secondly, the inner part of the medulla contains a peripheral nerve extending from the dental pulp into the apical papilla which stains positive for FAPα. SCAPs originate from neural crest cells (Chai et al., 2000) and have the capacity for neurogenic differentiation and thus could contribute to peripheral nerve growth (Kim et al., 2017). Interestingly, the region of the peripheral nerve resides a high concentration of round-shaped FAPα^+^/CD44^–^/vimentin^–^ cells. Since vimentin expression is absent in epithelial cells, we hypothesized that these cells could originate from an epithelial source such as HERS. A cytokeratin 19 staining revealed no positivity in this cell population excluding this assumption. Whether these cells belong to an intermediary phenotype of epithelial-mesenchymal cell transition (Klymkowsky and Savagner, 2009) requires further investigation. However, it is tempting to speculate that vimentin^–^/FAPα^+^ cells could be involved in maintaining the opening of the root canal during root maturation. The complete medulla is encapsulated by the cortex fibrosa which has a dense extracellular matrix composed of collagen type I. The majority of cells in this region are FAPα negative, vimentin positive and α-SMA negative, typical features of non-differentiated fibroblasts (Tillmanns et al., 2015). This region could provide mechanical support to the apical papilla. Furthermore, we observed FAPα positive odontoblasts inside dentin depositions within the medulla suggesting that FAPα positive SCAPs could act as precursors for odontogenic differentiation as shown before (He et al., 2014; Zhang et al., 2015; Wan et al., 2016). FAPα staining of dental pulp demonstrated that odontoblasts are indeed highly positive for FAPα which could be regulated by high expression of TGF-β receptors I and II (Smith et al., 1998). The function of FAPα in odontoblasts is currently not known but indicates a possible role in dentin formation. Within the dental pulp, FAPα positive cells were not that numerous compared to the medulla of the apical papilla. A considerable fraction of FAPα negative cells stained positive for vimentin but were negative for α-SMA matching the staining profile of non-differentiated fibroblasts. Whether FAPα expression discriminates between dental pulp fibroblasts and stem cells requires further investigation. We conclude that FAPα expression in combination with vimentin identifies heterogeneous cell populations in the apical papilla and dental pulp. However, we should note that there is a discrepancy between the heterogeneous FAPα staining in the tissue and the more homogenous staining in cell culture. This could be due to spontaneous differentiation of stem cells *ex vivo*. This assumption was made by the diffuse α-SMA staining in cultured cells which was absent in tissue. α-SMA expression is enhanced in culture due to the serum in the culture medium and the low compliance of the plastic culture substrate. A microarray study emphasized the role of the micro-environment on SCAPs gene expression as demonstrated by the significant number of differentially expressed genes between SCAPs in culture and from tissue (Diao et al., 2017). Finally, co-expression of FAPα and CXCR4 in the cytoplasmic extensions of cultured SCAPs and DPSCs suggests a joint role in cell migration. SDF-1α stimulates SCAPs chemo-attraction via activation of CXCR4 receptors (Liu et al., 2015) whereas FAPα facilitates cell migration via activation of RhoA GTPase activity as observed in BMSCs (Chung et al., 2014). An interplay between CXCR4 and FAPα could therefore enable chemo-attraction of SCAPs toward the region of root formation or DPSCs to the dentin border for odontoblast renewal. In addition, FAPα is not only localized on the plasma membrane but also at the nuclear region of dental stem cells. The role of nuclear FAPα is unknown but could share similarities with MMP-7 which after nuclear translocation enhances cell migration (Yingqiu et al., 2016). A further unknown factor is the molecular regulation of FAPα in mesenchymal stem cells and more specifically in dental stem cells. TGF-β1 could act as a possible regulator of FAPα expression since it is present as a latent form within the dentin matrix and it is a potent factor in stimulating odontoblast differentiation (Li et al., 2011). Moreover, it has been shown that the core promotor of FAPα mRNA transcription in humans contains TGF-β-responsive cis-regulatory elements (Zhang et al., 2010). However, other candidates which are reported to induce FAPα expression should not be ruled out including the transcription factors EGR1, TWIST1, and PARAXIS and a plethora of growth factors and cytokines regulating these downstream pathways (Busek et al., 2018).

## Conclusion

In conclusion, FAPα is considered a novel dental mesenchymal stem cell marker which could offer new future insights in dental root development and odontoblast function.

## Data Availability Statement

All datasets generated for this study are included in the article/Supplementary Material.

## Ethics Statement

This study protocol was approved by the Medical Ethical Committee of Hasselt University (protocol 13/0104U). All participants gave written informed consent in accordance with the Declaration of Helsinki except for cases where the participant was under the age of 16, where written informed consent was obtained from their legal guardians.

## Author Contributions

RD and IL designed the research. RD, PH, NS, and AB performed and analyzed the experiments. TV, YD, JR, EW, PG, MA, and IL contributed to the data interpretation and revised the manuscript. RD wrote the manuscript.

## Conflict of Interest

The authors declare that the research was conducted in the absence of any commercial or financial relationships that could be construed as a potential conflict of interest.
