# Supplementary material for: Dental Tissue and Stem Cells Revisited: New Insights From the Expression of Fibroblast Activation Protein-Alpha
Source: Front Cell Dev Biol. 2020 Jan 21;7:389. doi: 10.3389/fcell.2019.00389 (PMC6985075; doi:10.3389/fcell.2019.00389)
Supplement: Supplementary file 1 [file Presentation_1.pdf]

Supplementary Material

1 Supplementary Figures

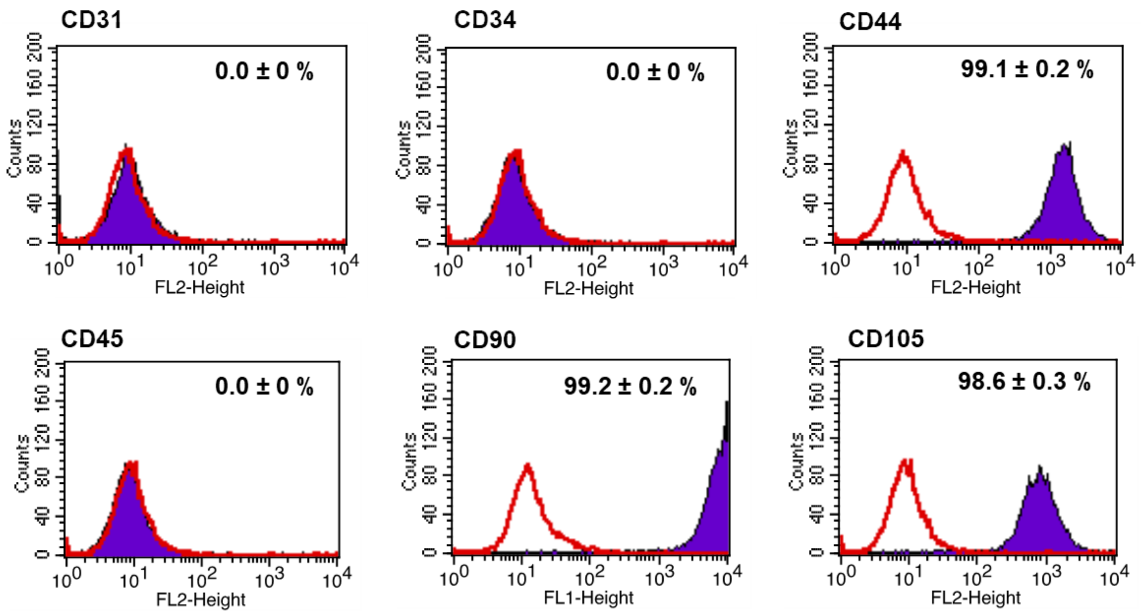

Supplemental Figure 1: Molecular characterization of dental follicle stem cells using FACS. Representative pictures of FACS analysis of cell surface markers CD31, CD34, CD44, CD45, CD90 and CD105. The purple histogram shows the immuno-stained cell population, while the red histogram indicates the appropriate isotype control. Values represents mean of at least 3 different patients ± S.E.M.

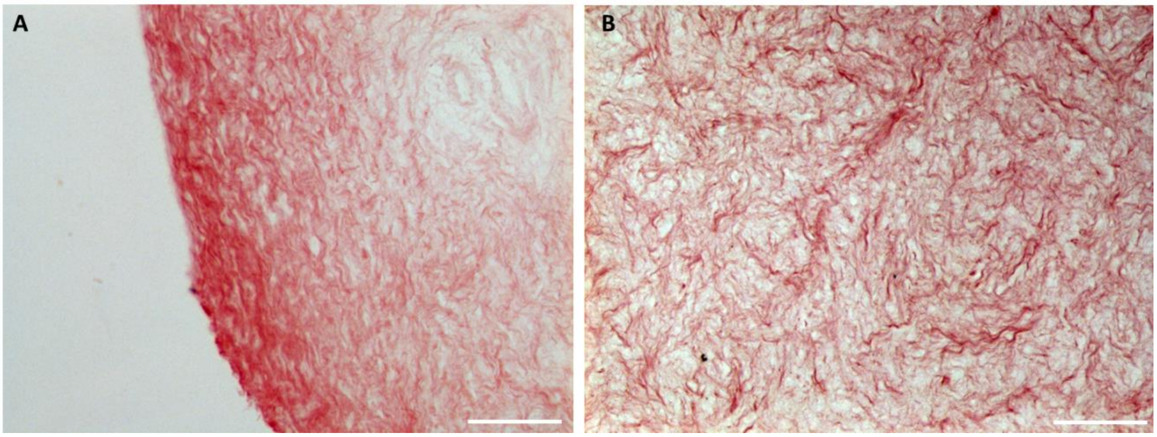

Supplemental Figure 2: Sirius red staining of the apical papilla showing collagen distribution in the cortex fibrosa and medulla. A. High concentration of aligned collagen in the cortex fibrosa. B. Unorganized collagen inside the medulla. Scale bars represent 50 µm.

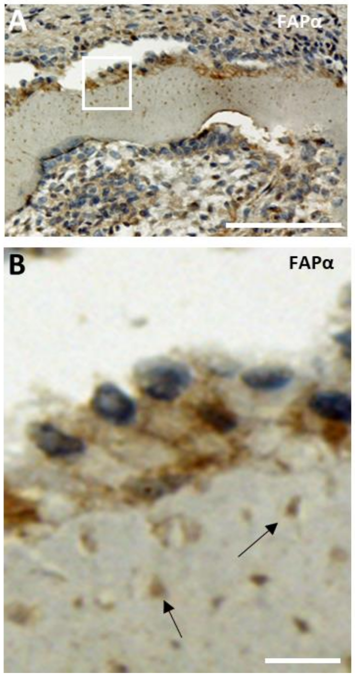

Supplemental Figure 3: FAPα expression in the dental nerve and in newly formed odontoblasts of the apical papilla. A. Newly formed dentin in the apical papilla. B. Higher magnification of the region indicated in A (white square) showing FAPα positive odontoblasts bordering the newly formed dentin. Note the FAPα positive odontoblast protrusions extending into the dental tubules. Scale bars represent 25 (B), 100 (A) µm.

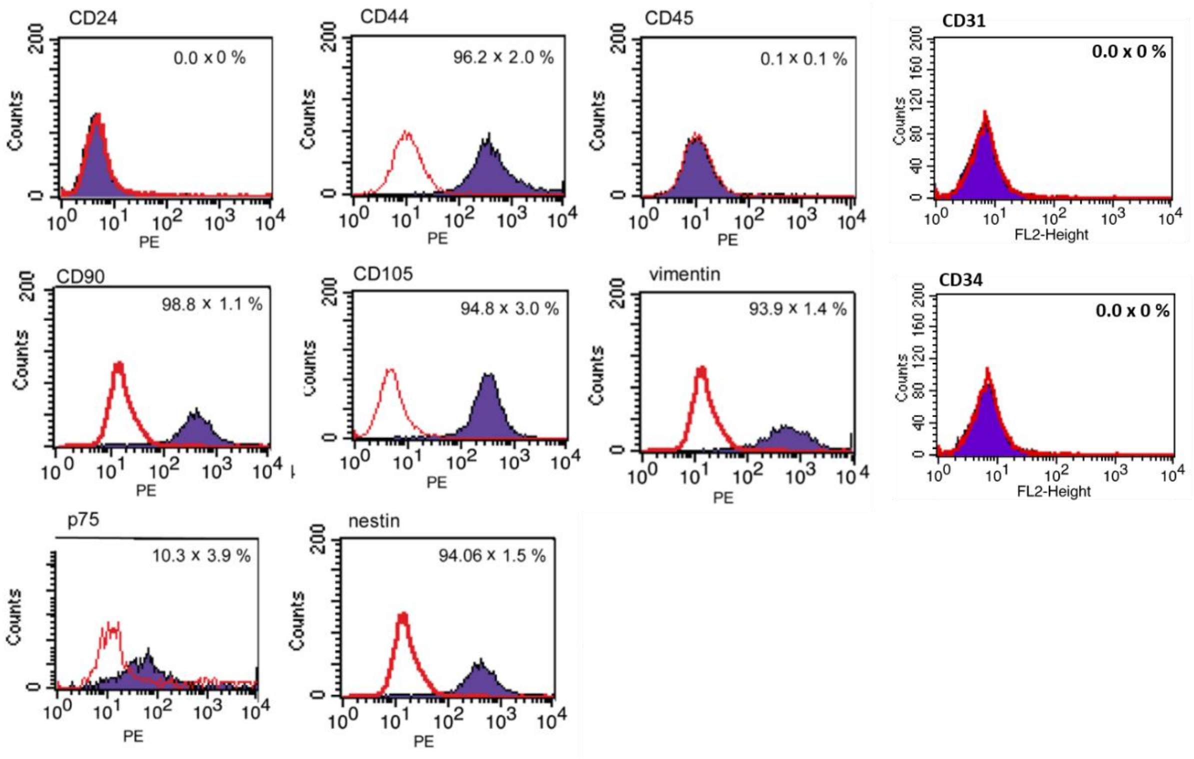

Supplemental Figure 4: Molecular characterization of SCAPs using FACS. Representative pictures of FACS analysis of cell surface markers CD24, CD44, CD45, CD31, CD90, CD105, CD34 and p75 and the intracellular proteins vimentin and nestin. The purple histogram shows the immuno-stained cell population, while the red histogram indicates the appropriate isotype control. Values represents mean of at least 3 different patients ± S.E.M. As is shown here, SCAP expressed mesenchymal stem cell markers CD44, CD90 and CD105, while the hematopoietic stem cell marker CD45 was not present. A subpopulation of SCAPs showed immune-reactivity for p75. Almost all cells expressed the neural precursor markers nestin and vimentin.
